# Early CCR2-dependent monocyte recruitment defines a critical neuroinflammatory window after subarachnoid hemorrhage

**DOI:** 10.3389/fimmu.2026.1888359

**Published:** 2026-08-14

**Authors:** Dong Su Kang, Seung Hyeok Seok, Seung-Hoon Lee, Yi Rang Na

**Affiliations:** 1 Translational Immunology Lab, Department of Transdisciplinary Medicine, Seoul National University Hospital, Seoul, Republic of Korea; 2 Immunology Core Facility, Department of Translational Research Center, Biomedical Research Institute, Seoul National University Hospital, Seoul, Republic of Korea; 3 Department of Biomedical Sciences, Seoul National University College of Medicine, Seoul, Republic of Korea; 4 Macrophage Lab, Department of Microbiology and Immunology, Seoul National University College of Medicine, Seoul, Republic of Korea; 5 Institute of Endemic Diseases, Seoul National University Medical Research Center (SNUMRC), Seoul, Republic of Korea; 6 Cancer Research Institute, Seoul National University, Seoul, Republic of Korea; 7 Cenyxbiotech Inc., Seoul, Republic of Korea; 8 Department of Neurology, Seoul National University Hospital, Seoul, Republic of Korea; 9 Department of Medicine, Seoul National University College of Medicine, Seoul, Republic of Korea

**Keywords:** CCR2^+^monocyte, early brain injury, neuroinflammation, subarachnoid hemorrhage, therapeutic window

## Abstract

Subarachnoid hemorrhage (SAH) remains a devastating cerebrovascular disease with limited therapies targeting secondary neurological injury. Increasing evidence suggests that the early brain injury (EBI) period within the first 72h after SAH represents a critical therapeutic window characterized by diffuse neurovascular and inflammatory activation within the cerebrospinal fluid (CSF) and meningeal compartments. Although resident microglial responses during EBI have been extensively investigated, the contribution of infiltrating CCR2-dependent monocytes to inflammatory amplification and secondary neuronal injury during this acute phase remains incompletely defined. Here, we investigated the temporal role of CCR2-dependent monocyte recruitment during EBI following experimental SAH. Using a murine endovascular perforation model, we evaluated outcomes in CCR2-deficient mice and wild-type mice treated with a pharmacologic CCR2 antagonist at distinct post-SAH timepoints. CCR2 deletion or early pharmacologic inhibition significantly reduced Ly6C^hi^ monocyte infiltration during EBI, attenuated CSF TNFα and IL-6 levels, reduced delayed neuronal injury within the motor cortex, and improved neurological recovery after SAH. Importantly, therapeutic benefit was strongly time dependent, as delayed CCR2 inhibition after the EBI period failed to improve long-term motor outcomes. To investigate mechanisms underlying monocyte-associated neurotoxicity, human cortical neurons were exposed to hemin-induced hemorrhagic stress in the presence of THP-1 monocytes. Monocyte co-culture exacerbated neuronal injury and increased TNFα production, whereas TNFα neutralization partially restored neuronal viability under severe hemorrhagic stress conditions. Together, these findings identify early CCR2-dependent monocyte recruitment temporally linked to inflammatory amplification and secondary neuronal injury during the EBI phase following SAH. Our results support early, time-sensitive CCR2 modulation as a potential therapeutic strategy for improving neurological recovery after SAH.

## Introduction

Subarachnoid hemorrhage (SAH), commonly caused by aneurysmal rupture, is a devastating cerebrovascular disease with high mortality and long-term neurological disability (1–3). Although advances in surgical and endovascular management have improved survival rates over recent decades (4), many patients continue to experience substantial secondary complications such as vasospasm, delayed cerebral ischemia (DCI), and long-term motor deficits (3, 5, 6). While current therapies partially address vascular complications (7, 8), effective strategies for ameliorating neurological deficits are limited.

The early brain injury (EBI) period, defined as the first 72h following SAH, is increasingly recognized as a critical determinant of neurological outcome (9–11). During this phase, pathological processes, including elevated intracranial pressure (ICP), endothelial injury, microvascular dysfunction, and neuroinflammation contribute to secondary brain injury (12–17). Unlike intracerebral hemorrhage, SAH exposes the subarachnoid and cerebrospinal fluid (CSF) compartments to diffuse blood breakdown products, promoting widespread neuroimmune activation during EBI. In addition to resident immune responses, peripheral myeloid cells are recruited to the hemorrhagic CNS environment and may further amplify inflammatory signaling (17–21). Among these infiltrating immune populations, classical monocytes represent a major subset recruited through C-C Chemokine Ligand 2 (CCL2 or MCP-1)-CCR2 signaling (22). Although infiltrating monocytes have been observed following SAH, the temporal contribution of CCR2-dependent monocyte recruitment to inflammatory amplification and secondary neuronal injury during the EBI period remains poorly defined.

In this study, we investigated whether CCR2-dependent monocyte recruitment contributes to secondary injury progression during the EBI phase following SAH. Using a murine SAH model with both genetic and pharmacologic inhibition of CCR2 signaling, we evaluated whether suppression of monocyte recruitment could attenuate secondary inflammatory responses, neuronal injury, and neurological recovery. We found that early suppression of CCR2-dependent monocyte infiltration attenuated neuroinflammatory responses, reduced neuronal injury, and improved functional recovery after SAH, whereas delayed intervention failed to provide comparable benefit. Together, these findings identify CCR2-dependent monocyte recruitment as a temporally critical contributor to secondary injury progression during EBI and suggest that precisely timed immune modulation may represent a potential therapeutic strategy following SAH.

## Materials and methods

### Animals

All animal experimentations and animal care were carried out following the guidelines approved by the Institutional Animal Care and Use Committee (IACUC) of Seoul National University (IACUC No. SNU-240220-1). Mice were housed in the animal care facility under 12h light/dark cycle with access to food and water. All possible measures were taken to minimize suffering and reduce the number of animals used in this experiment. All mice used for modeling were within the age of 12 to 14 weeks for MCA perforation modeling. All mice had a C57BL/6 background, and the following strain was used as WT or control. C57BL/6-Tg (hCD68-EGFP)1Drg/J, Jackson #026827 was used for meningeal whole-mount imaging. B6.129S4-Ccr2^tm1Ifc^/J, Jackson #004999 Homozygotes were selected for the use of all CCR2^−/−^ SAH models.

### SAH modeling

The MCA perforation SAH model was established by following the protocol from Du et al., 2016 (23) with modifications. All mice prior to surgery were under anesthesia using 4% isoflurane in 30% oxygen and were maintained using the anesthesia systems (Vetflo). Mice were laid in a supine position with exposed necks, and skin was opened near, slightly off the midline toward the ipsilateral side of the targeted side. Connective tissues were bluntly dissected through until the external, internal, and common carotid arteries (ECA, ICA, and CCA, respectively) were identified. Using a black silk 6–0 filament, ECA was ligated, and CCA was temporarily ligated with silk line and hemostats. Blue Nylon (5–0) was used for perforation, which entered from ECA, traveling through ICA until it reached the MCA. After the MCA perforation, all mice after SAH modeling were placed in a cage with infrared heating for 20 to 30 min to prevent hypothermia. For mice with PLX treatment, mice were given PLX3397-infused Purina chow (600 ppm) for 2 weeks before SAH modeling was performed.

Hemorrhage severity was assessed for animals that were analyzed for FCM and histology using a modified Sugawara grading approach (24). Animals with a modified score < 6, were excluded due to insufficient SAH severity.

### Meningeal wholemount

For meningeal whole mount to confirm immune cell infiltration, we followed the protocol in accordance with Louveau et al., 2019 (25), with slight modifications. First, Skull cap of hCD68-GFP mice were harvested after CO_2_ euthanization. Then, Skull caps were fixed in 4% PFA in PBS overnight at 4 °C. Then under a petri dish and a dissecting microscope, meninges were carefully detached. Detached meninges were washed with PBS for 5 min, and for protocol for immunofluorescence staining was performed.

### Flow cytometry

Flow cytometry was performed for immune cell profiling. Mice were anesthetized and perfused with PBS prior to sampling. Prior to the brain extraction, CSF was obtained from their cisterna magna using an insulin syringe (BD). The brainstem and cerebellum were removed, and the brain was minced to <1 mm in collagenase V (Sigma, St. Louis, MO, USA, C9263, 1 mg/ml) infused with 5% FBS in PBS with DNase (100 units), and incubated in a 5% CO_2_ incubator at 37°C for 20 min. Then, EDTA was given to neutralize digestion. The samples were filtered through 70 μm cell strainer into a 50-ml tube. Cell pellets were collected by centrifugation of 1,300 *rpm* × 5 min. Myelin & neurons were removed using 30% Percoll™ density gradient media (Cytiva) in 1 × PBS. Then, 6 ml of ammonium-chloride-potassium (ACK) lysing buffer (4M potassium bicarbonate, 0.5M ammonium chloride in ultrapure water) was applied for 5 min to remove any remaining red blood cells. The ACK buffer was stopped by adding 1 × PBS up to 50 ml, then was centrifuged at 1,300 *rpm* for 5 min. The following antibodies were diluted in a 1:500 ratio in FCM buffer (5% FBS in PBS, filtered, with 0.1% sodium azide) and stained for 20 min on ice: CD45 BUV395, CD11b V450, Ly6G PE, CX3CR1 APC-Cy7, and Ly6C BV786 (all antibodies were purchased from BioLegend). DAPI was stained separately at a 1:5000 dilution for cell viability. Samples were washed, resuspended in FCM buffer (200 µl), and moved to an FCM tube before flow cytometry data acquisition in FACS Symphony A5 (BD Biosciences). All data were gated and analyzed using FlowJo_v10.10.

### Cytometric bead array

CBA was performed using materials from BD™ CBA Mouse Inflammation Kit (BD Biosciences, 552364), detecting KC, IL-1β, IL-6, IL-10, and TNFα in accordance with the product guidelines. The staining process was the same as CBA, with exceptions to tissue preparation. Tissues were put into an Eppendorf tube with 300 µl of NP40 lysis buffer (Invitrogen, 289375-000) with a 1:500 dilution of protease and phosphatase inhibitor (GenDEPOT). Metal beads were inserted into a tube containing tissues, and TissueLyserII (QIAGEN) was used to lyse the tissues. Chloroform is added (200 µl) to the lysate solution and centrifuged at 13,000 *rpm* for 15 min at 4 °C. The top forming layer was extracted, containing proteins. For the CSF sample, this step was skipped. Then all assays were performed according to product guidelines. Data was obtained using a FACSymphony A5 flow cytometer. Raw was analyzed using FCAP Array V3 Software and was quantified into graphs using GraphPad Prism 10.1.

### Neurological score, survival curve, and long-term behavioral test

For neurological assessment, Modified Garcia scoring (26) was performed on Post Operative Day (POD) 1, 3, and 5 with modifications on the table (**Supplementary Table 1**). Lateral turning was excluded from the assessment due to potential bias from surgical sutures during the acute-postoperative period. Animals displaying neurological score above 15 were excluded from further a neurological assessments for not meeting the pre-defined SAH severity threshold (24). Animals that died before POD 5 were not available for neurological assessment at later time points and were excluded in final data analysis. Mice receiving CCR2 inhibitor treatment were only at POD1 to evaluate the immediate effects of treatment.

All mice for motor assessment were given time for recovery until POD 28. Each mouse was checked daily for survival for up to 2 weeks, as mortality is highest during this period (27). For subsequent long-term behavioral tests, animals exhibiting a modified Garcia score <5 at POD 5 were not included, as they rarely survived beyond POD 7 regardless of genotype, and those that survived were unable to reliably perform motor behavioral tasks due to profound neurological deficits. On POD 28, mice were moved to a behavioral test room 1h prior to the rotarod test to get acclimated to the environment. Each mouse was then placed onto the rotarod for a familiarization trial before formal testing. The rotarod test was performed with acceleration at a rate of 4 rpm/min. Latency and final RPM before falling were recorded and analyzed.

### TUNEL staining

The *in-situ* Cell Death Detection Kit, POD (Roche, 12156792910), was used for neuronal cell death. To assess secondary neuronal damage, we selected mice that had blood present in the ipsilateral hemisphere of the perforation site at POD 3. All harvested mouse brains were fixed in 4% PFA in PBS overnight and were made into FFPE. Each section obtained was 6–8 μm. We located area of interest using the Allen Brain Institute 3D mouse brain atlas (see 28) to obtain a section containing motor cortex. To obtain the section of interest, we trimmed from the prefrontal cortex to 460 to 480 μm. Once the lateral ventricles were shaped in accordance with the previously viewed image, we obtained sections within a 20 μm difference. For obtaining a section with a hippocampus, we proceeded with an additional 400 μm trimming, repeating the procedure.

Samples were mounted in Permount mounting medium (Fisher Chemical). All images were slide-scanned using Leica Biosystems-Aperio and were analyzed and obtained using QuPath 0.5.0 software.

### Immunofluorescence staining

To visualize CCR2, we performed immunofluorescent staining on the tissue section or meningeal whole mount mentioned above. First, samples on slides were permeabilized by incubating with 0.3% Triton-X in 1 × PBS. Then, samples were washed with PBS twice, 5 min each. After washing, samples were blocked using 3% BSA and 0.3% Triton-X in PBS for 30 min. The CCR2 antibody was diluted 1:200 (Abcam, ab273050) in antibody dilution solution (1% BSA, 0.1% Triton-X in PBS), and slides were incubated in it overnight at 4 °C. Next, slides were washed three times with PBS for 5 mib each, then were incubated with Alexa 555 anti-rabbit for visualization (Invitrogen, A-21428) diluted at 1:2000 in antibody dilution solution for 1h at room temperature. Then slides were washed with PBS twice and incubated with PBS containing DAPI (1:2000) for 10 min. Slides were washed with PBS twice again and were mounted under Vecta-shield antifade mounting medium (Vector Lab., H-1900-10). For image acquisition, a Leica TCS SP-5 confocal microscope was used for brain section images. For wholemount imaging, we used Benchtop Confocal BC43 (Oxford instrument, Andor) for the entire wholemount scanned image. All images were then acquired with ImageJ or IMARIS software.

For visualizing TUNEL-positive neuron staining, the *in-situ* Cell Death Detection Kit was used again without a POD converter. After the TUNEL staining, we used anti-NeuN (Abcam, ab104225) diluted 1:200 for staining.

### CCR2 inhibition

To evaluate the effect of CCR2 inhibition, SAH was induced in C57BL/6 mice. Mice received a single intraperitoneal injection of RS504393 (MedChemExpress, HY-15418; 9 mg/kg), dissolved in 5% DMSO in PBS at either 1h (Early RS) or 72h (Late RS) after SAH induction. The selected dose was based on *Tian* et al. *(2022)* (27), which demonstrated effective *in vivo* CCR2 blockade. Vehicle-treated mice received an equivalent volume of 5% DMSO in PBS. In the present study, RS504393 was administered as a single dose to target CCR2-dependent monocyte recruitment during defined post-SAH time windows rather than to achieve continuous CCR2 inhibition throughout the recovery period.

For flow cytometric validation of monocyte infiltration, the Sham, SAH + Vehicle, and SAH + Early RS groups underwent neurological assessment using the modified Garcia score 24h after surgery. Subsequently, brains were harvested for FCM (*n* = 4–5 per group) and analysis to assess infiltrating CD45^hi^ myeloid cells and monocytes.

For long-term functional recovery experiments, the same experimental groups were used with the addition of an SAH + Late RS group. Animals were followed for 4 weeks after SAH induction, and surviving mice underwent rotarod testing as described in the Behavioral Assessment section (*n* = 4 per group). One mouse from the vehicle group reached a humane endpoint before rotarod testing and was euthanized; therefore, it was excluded from behavioral analysis.

### Statistical analysis

All statistical analyses were performed using GraphPad Prism 10.1. Longitudinal Garcia score data were analyzed using two-way repeated-measures analysis of variance (ANOVA) with Geisser–Greenhouse correction, followed by Holm-Šídák’s multiple-comparisons test. Motor performance, single-time-point Garcia scores, and flow cytometry datasets involving multiple group comparisons were analyzed using one-way ANOVA followed by Tukey’s multiple-comparisons test. *In vitro* experiments were analyzed using ordinary two-way ANOVA. Survival curves were analyzed using the Mantel-Cox (log-rank) test. Corresponding p-values are presented within the figures. Detailed statistics for relevant figures can be found in the supplementary table (**Supplementary Table 2**). All quantified data are presented as mean ± SEM. Statistical significance was defined as *p* < 0.05.

### Cell culture and *in-vitro* experiment design

Human cortical neurons (HCN) and the human monocytic cell line THP-1 were maintained under standard culture conditions at 37°C in a humidified atmosphere containing 5% CO_2_. HCN cells were cultured in Dulbecco’s Modified Eagle Medium (DMEM) supplemented with 10% fetal bovine serum (FBS) and 1% penicillin–streptomycin. THP-1 cells were cultured in RPMI-1640 supplemented with 10% heat-inactivated FBS and 1% penicillin–streptomycin. All cells were routinely tested and confirmed to be free of mycoplasma contamination.

Prior to hemin treatment, HCN cells were seeded into black-walled 96-well plates at a density of 1 × 10^4^ cells/well and allowed to adhere for at least 18h. For HCN-only control wells, cells were seeded at 1.5 × 10^4^ cells/well to match total cell numbers used in co-culture conditions.

To model blood breakdown product–induced cellular stress relevant to SAH, hemin (MedChemExpress, HY-19424) was dissolved in NaOH according to the manufacturer’s instructions to generate a 5 mM stock solution. Aliquots were stored at −80°C until use. The following experimental groups were included: (1) HCN control, (2) THP-1 control, (3) HCN + THP-1 co-culture (Co), (4) HCN + hemin, (5) Co + hemin, (6) Co + hemin + CCR2 inhibitor, and (7) Co + hemin + TNFα inhibitor.

Hemin was diluted to final concentrations of 10, 50, 80, or 150 µM in a 1:1 mixture of DMEM and RPMI-1640. Vehicle controls received NaOH at equivalent dilutions. Depending on the experimental group, culture media were supplemented with vehicle (DMSO), the TNFα inhibitor C-87 (10 µM; MedChemExpress, HY-100735), or the CCR2 antagonist RS504393 (10 µM). Conditioned media were used to resuspend THP-1 cells, which were then added to HCN cultures at a density of 5 × 10^3^ cells/well to establish co-culture conditions mimicking the SAH environment. For THP-1-only control wells, cells were seeded at 1.5 × 10^4^ cells/well to match total cell numbers across conditions.

Cell viability was assessed using the CellTiter-Glo^®^ 2.0 Cell Viability Assay (Promega, G9241) according to the manufacturer’s instructions. Luminescence was measured using a BioTek Synergy™ Mx microplate reader, and all data were analyzed using GraphPad Prism.

The supernatant was also partially collected after 6h of hemin incubation in each group at vehicle, 50 and 150 µM hemin, and TNFα concentration was determined using the Human TNFα ELISA DuoSet (R&D Systems, DY210).

## Results

### CCR2^+^ monocyte recruitment peaks during the early brain injury period after SAH

**Figure 1:**
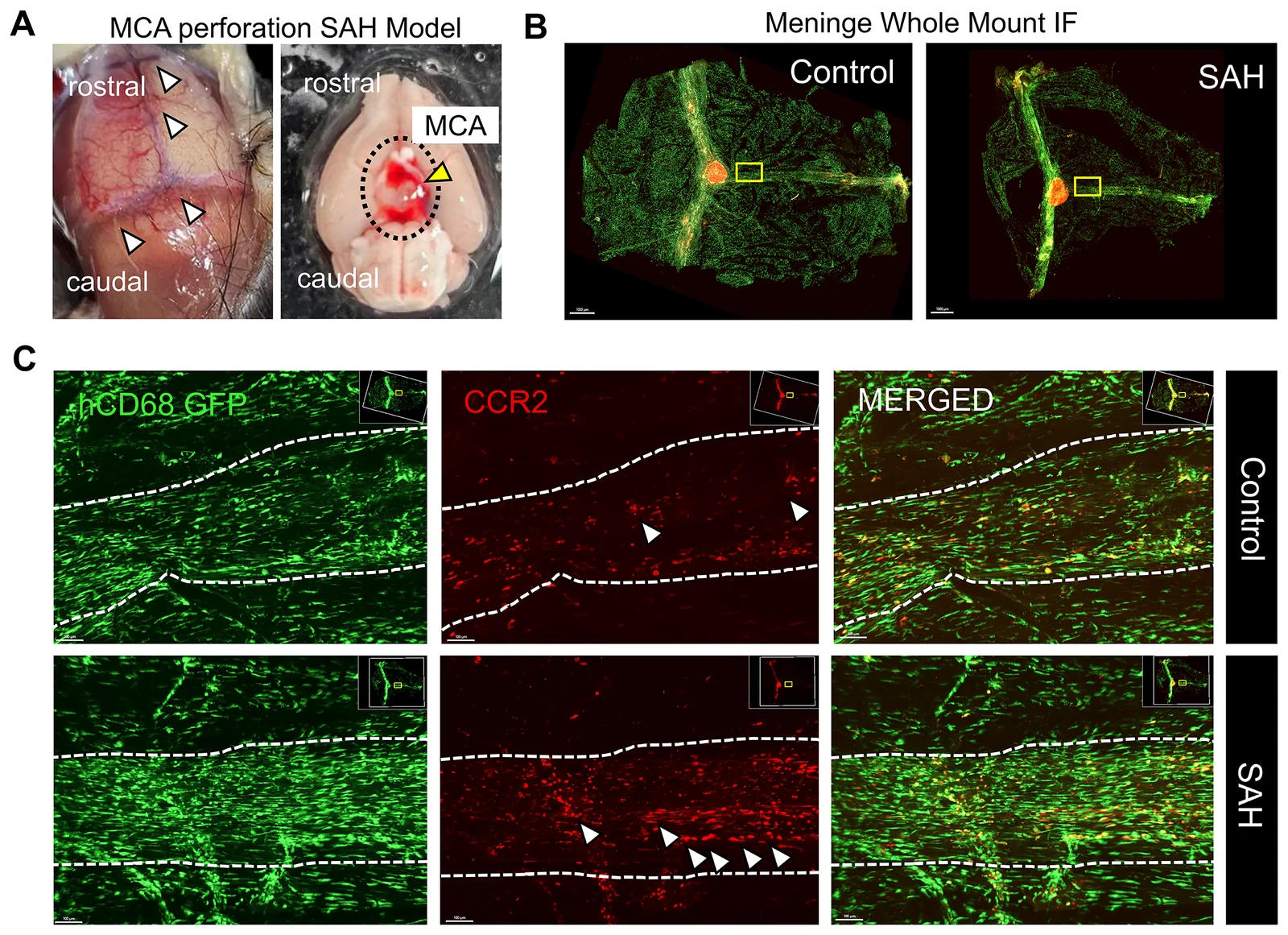
Infiltrating CCR2+ cells increase in the meningeal layer during early EBI of SAH. **(A)** Representative image of SAH mice model confirming hemorrhage in meningeal layer before harvesting brain (left) and bleeding remains after perfusion (right, black dotted circle: Circle of Willis, Yellow arrow: MCA perforation site). **(B)** Meningeal wholemount scanned image of Control and SAH hCD68-GFP mice. Scale bar = 1mm. Yellow square represents zoomed in area of (**Figure 1C**). **(C)** Images of 10× zoom in of SAH and control at superior sagittal sinus, demarcated by the white dotted line. Images of GFP^+^ myeloid cells (Green), CCR2 (red), and merged images are shown. White arrows indicate CCR2^+^ cells. EBI, early brain injury; SAH, subarachnoid hemorrhage; MCA, middle cerebral artery; CCR2, C; Ctrl, Control. *Scale bar =100 μm; n = 3 per group*.

To confirm the increasing CCR2^+^ monocyte recruitment during the EBI period of SAH mice, we established an MCA perforation SAH mice model. We confirmed the presence of hemorrhage on the hemisphere ipsilateral to the perforation site, which remained even after perfusion near the MCA (**Figure 1A**). After establishing the SAH mice model, we questioned if a monocyte population increase was observable within the meninges during the EBI period, as it has been highlighted as an immune hub for neuroimmune surveillance (29); while direct evidence for increased neutrophils within the meninges has been present, evidence showing monocyte recruitment during EBI period has not been well-observed. Using hCD68-GFP reporter mice, which show GFP expression in monocytes and macrophages, we determined whether monocyte were present within the meninges during early EBI. Generally, GFP signals were evenly distributed throughout the leptomeninges in control mice (**Figure 1B**). However, after the first 24h (Post Operative Day 1, POD 1) of SAH, the GFP signal was increasingly concentrated within sinus regions, a well-known site for immune cell recruitment during neuroinflammation (30, 31). Notably, we saw a significant increase in the Superior Sagittal Sinus (SSS) (**Figure 1C**). We also confirmed an increase in CCR2^+^ signals, along with CCR2^+^ GFP^+^ double positive cells, suggesting that monocytes are heavily recruited into the meningeal layers within the early EBI period of SAH.

### Decreased immune infiltration during EBI of the SAH mice models in CCR2^−/−^ mice

**Figure 2:**
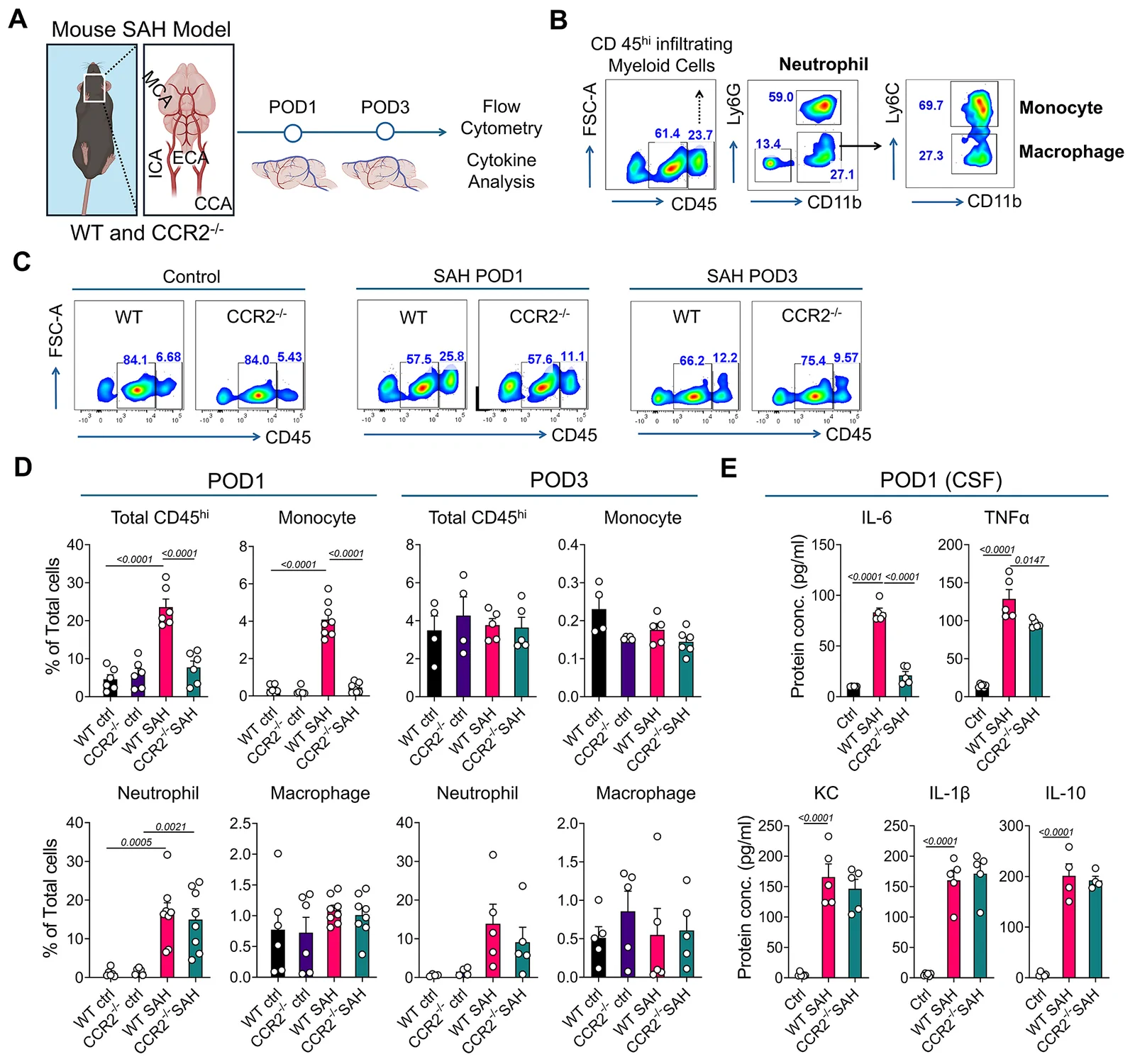
CCR2^−/−^ SAH mice models reduced immune cell infiltration, particularly Ly6C^hi^ monocytes, and reduced CSF IL-6 and TNFα cytokines. **(A)** Schematic depiction of SAH modeling to flow cytometry performance. **(B)** Gating strategy of Infiltrating myeloid cells, and specific innate immune cells. **(C)** Representative population difference in CD45^hi^ population in flow cytometry data. **(D)** Quantification of cells from flow cytometry data analyzed at POD1 (top) and POD3 (bottom). **(E)** CBA data quantified for determining cytokine concentrations within CSF. POD, post-operative day; CSF, cerebro spinal fluid. Panels **(D, E)** are presented as mean ± SEM. POD1 n = 6 per ctrl, n = 8 per SAH group. POD 3 n = 4 per ctrl n = 5 per SAH group. One-way ANOVA with Tukey’s multiple comparisons test was performed. Significant p-values are indicated in the graph.

After establishing the robust early recruitment of CCR2^+^ monocytes during the EBI period, we next investigated whether CCR2 deletion alters the broader immune cell profile during this acute phase. CCR2^−/−^ and WT mice were subjected to experimental SAH, and immune populations were examined using flow cytometry across the first 72h post-injury (**Figures 2A, B**). At POD 1, CCR2**^−/−^** SAH mice exhibited approximately a 50% reduction in total infiltrating CD45^hi^ immune cells compared to WT SAH mice, which was no longer evident by POD 3 (**Figure 2C**). Subpopulation analysis showed a significant reduction in CD45^hi^ CD11b^+^ Ly6C^hi^ monocytes in CCR2**^−/−^** mice during POD1, whereas neutrophil (CD11b^+^ Ly6G^+^) and macrophage (CD45^hi^CD11b^+^Ly6C^−^) populations were not significantly altered (**Figure 2D**). Together, this indicates that CCR2 deletion primarily affects monocyte recruitment following SAH.

We next performed cytokine analysis during POD 1 in brain lysates and CSF to determine whether reduced monocyte infiltration altered inflammatory signaling. In brain tissue, when compared with respective controls, SAH induced elevations of cytokines in both genotypes. CCR2**^−/−^** mice had significantly elevated IL-10, while WT mice had elevated TNFα levels following SAH (**Supplementary Figure 1A**). No significant genotype-dependent differences were observed for TNFα, IL-1β, or IL-10 when directly comparing WT and CCR2^−/−^ SAH groups, whereas CCR2**^−/−^** SAH mice had significantly reduced CXCL1 (KC) and IL-6 concentrations compared to WT SAH mice. In CSF, cytokine levels were substantially elevated after SAH, consistent with compartmentalized inflammatory responses (**Figure 2E**). We observed significantly reduced TNFα and IL-6 in CCR2**^−/−^** SAH mice compared to WT SAH mice, whereas CXCL1 concentrations did not show a difference.

Collectively, these findings suggest that CCR2 deletion reduces Ly6C^hi^ monocyte recruitment rather than broadly suppressing myeloid cell infiltration during the EBI period and coincides with reduced pro-inflammatory cytokine signaling within the CSF.

### CCR2^−/−^ SAH mice exhibit improved motor recovery compared with WT

**Figure 3:**
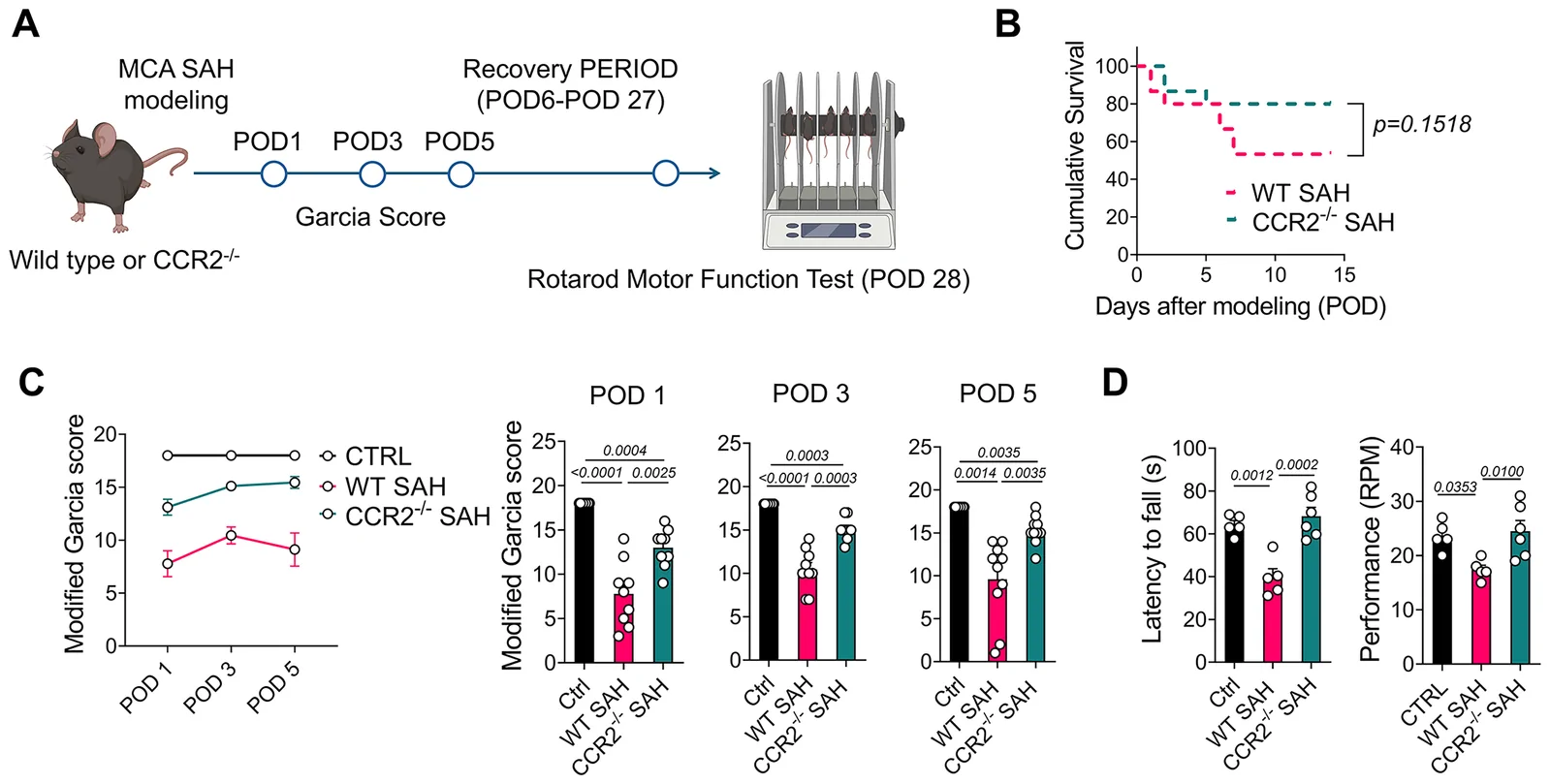
CCR2−/− SAH mice model has better neurological recovery & long-term motor performance. **(A)** Schematic of all neurological and behavioral tests. **(B)** Survival curve determined up to POD 14. **(C)** Modified Garcia score chart with combined days to show changing average score (left) and individual day (right). **(D)** Rotarod result on POD 28 with endurance (Left) and motor performance (Right). *For B, N = 8 per group*, Mantel-Cox (log-rank) test performed. For C, n = 9 per group. Two-way RM-ANOVA, with Holm-Šídák’s multiple comparisons test performed. For D, n = 8–9 per SAH group and n = 5 for controls. Mice were excluded for death or extreme motor deficits, unable to perform motor test. Final n = 5–6 per group. One-way ANOVA with Tukey’s multiple comparisons test was performed. Panels **(C, D)** are presented as mean ± SEM. Significant p-values are indicated in the graph.

To determine whether CCR2 deletion improved functional recovery after SAH, we assessed short- and long-term neurological outcomes (**Figure 3A**; **Supplementary Figure 2A**). Previous studies show that surviving SAH mice show neurological differences in both short-term (POD 3 to 7) and long-term recovery (up to POD 28) (32, 33). Initially, we determined whether CCR2 deletion is associated with survival rate. Although CCR2**^−/−^** mice had higher cumulative survival (80%) compared to WT mice (53%), there was no significant difference in survival rate over the first two weeks (*p = 0.15*; **Figure 3B**). With no difference in survival, we examined neurological outcomes. CCR2**^−/−^** SAH mice demonstrated significantly better modified Garcia scores than WT SAH mice on POD 1, 3, and 5, with most differences observed in the climbing sub-score (**Figure 3C**; **Supplementary Video S1**). We also tested the microglia-depleted WT SAH mouse model using PLX chow, which showed no recovery compared to WT SAH (**Supplementary Figure 2A**), suggesting that microglial depletion does not lead to functional benefit in this model. At POD 28, motor performance on the rotarod was significantly better for the CCR2**^−/−^** SAH group, with performance comparable to control groups (**Figure 3D**; **Supplementary Figures 2B**-**S2D**). In contrast, the Morris water maze revealed no significant difference in spatial learning or memory between groups. Together, these findings suggest that CCR2 deletion primarily improves motor and functional recovery following SAH, with no detectable benefit in cognitive performance under the conditions examined.

### CCR2^−/−^ SAH mice exhibit reduced neuronal cell death compared to WT SAH mice

**Figure 4:**
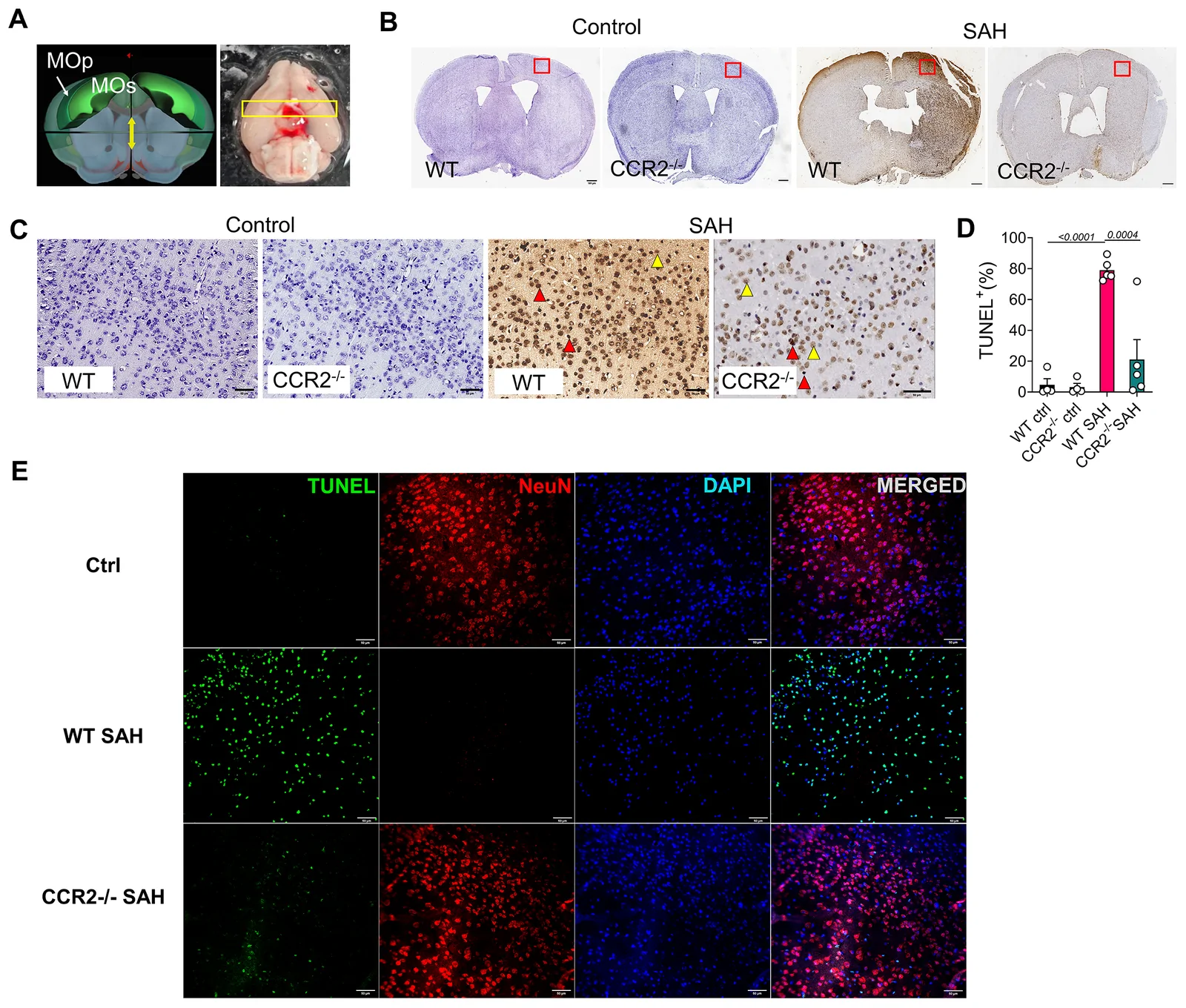
TUNEL Assay shows CCR2−/− SAH mice model has reduced neuronal cell death. **(A)** Selected area for TUNEL staining. We used *ALLEN 3D BRAIN ATLAS* (left). For accurate plane of section where motor cortex was present, indicated by the yellow box drawn on actual sample (right). MOp, Primary Motor area; MOs, Secondary Motor area. **(B)** Representative image of TUNEL staining for each group. Red squares indicate the zoomed in region of motor cortex shown in figure **(C)** zoomed in motor cortex area of the slide scan in Figure **(B)** Red arrows indicate examples of TUNEL^hi^ cells, while yellow arrows indicate examples of TUNEL^low^ cells. **(D)** Quantified TUNEL staining with total count proportion (left) and classified staining (right). **(E)** Representative Immunofluorescent staining of TUNEL with anti NeuN to determine neuron-specific cell death. Panel **(B–E)**, N = 4 for each Ctrl, N = 5 for each SAH group. Panel **(B)**, Scale bar = 500 μm. Panels **(C, E)**, Scale bar = 50 μm. Panels **(D)** is presented as mean ± SEM, and one-way ANOVA with Tukey’s multiple comparisons test was performed. Significant p-values are indicated in the graph.

Next, to assess whether CCR2 deficiency reduces secondary neuronal injury, we analyzed mice with confirmed ipsilateral hemorrhage at POD 3. Given that SAH is associated with secondary neuronal damage and delayed cerebral infarction (34), we assessed neuronal cell death late in POD 3, corresponding to the late EBI period during which neuronal death is expected to have occurred (35). Using the Allen Brain Atlas (28), we identified cortical sections containing the motor cortex and performed a TUNEL assay to determine cell death (**Figures 4A, B**).

CCR2**^−/−^** SAH mice exhibited a significant reduction in both TUNEL positive intensity and area compared to WT SAH mice, with a similar anatomical distribution pattern. IBA1 immunostaining did not reveal any significant differences in microglial number or morphology between the two groups (**Supplementary Figure 3A**). We observed the most pronounced differences in TUNEL signals within the motor cortex, consistent with motor deficits observed in behavioral testing (**Figure 4C**). In contrast, differences between dentate gyri (DGs) were barely noticeable with minimal TUNEL signals in both groups, suggesting region-specific vulnerability during the late EBI period in this model (**Supplementary Figure 3B**). Quantitative analysis revealed a significant reduction in total TUNEL-positive cells in CCR2**^−/−^** SAH mice compared to WT SAH mice (**Figure 4D**), corresponding to an approximate 75% decrease.

To determine whether TUNEL positivity was associated with neuronal cell death, we performed co-immunostaining with NeuN (**Figure 4E**). Within the cortex ipsilateral to MCA perforation, WT SAH mice exhibited substantial loss of NeuN immunoreactivity accompanied by strong TUNEL positivity, consistent with extensive neuronal degeneration. In contrast, CCR2^−/−^ SAH mice showed a reduced TUNEL^+^ signal and preservation of NeuN immunoreactivity in the same region, suggesting attenuation of secondary neuronal injury. As extensive neuronal damage in the severely affected cortical regions resulted in dense TUNEL labeling and loss of NeuN signal, reliable assessment of TUNEL/NeuN co-localization was not feasible in these areas. Moreover, loss of NeuN immunoreactivity following brain injury may reflect altered NeuN antigenicity rather than complete neuronal loss, necessitating cautious interpretation of NeuN staining alone (36). Therefore, co-localization analysis was performed in adjacent cortical regions where individual neuronal profiles could be reliably identified with NeuN along with TUNEL signal (**Supplementary Figures 4A**, **B**). Quantifications revealed that over 90% of NeuN+ neurons in WT SAH mice were TUNEL positive, whereas this proportion was reduced to approximately 60% in CCR2^−/−^ SAH mice. Notably, microglial depletion prior to SAH resulted in widespread TUNEL positivity despite partial preservation of NeuN immunoreactivity (**Supplementary Figure 3C**), consistent with increased neuronal vulnerability following global microglial depletion. Together, these findings demonstrate that neuronal cell death is a major component of secondary injury following SAH and CCR2 deficiency attenuates secondary neuronal injury.

### Pharmacologic CCR2 inhibition during the early EBI period improves motor recovery in WT SAH mice

**Figure 5:**
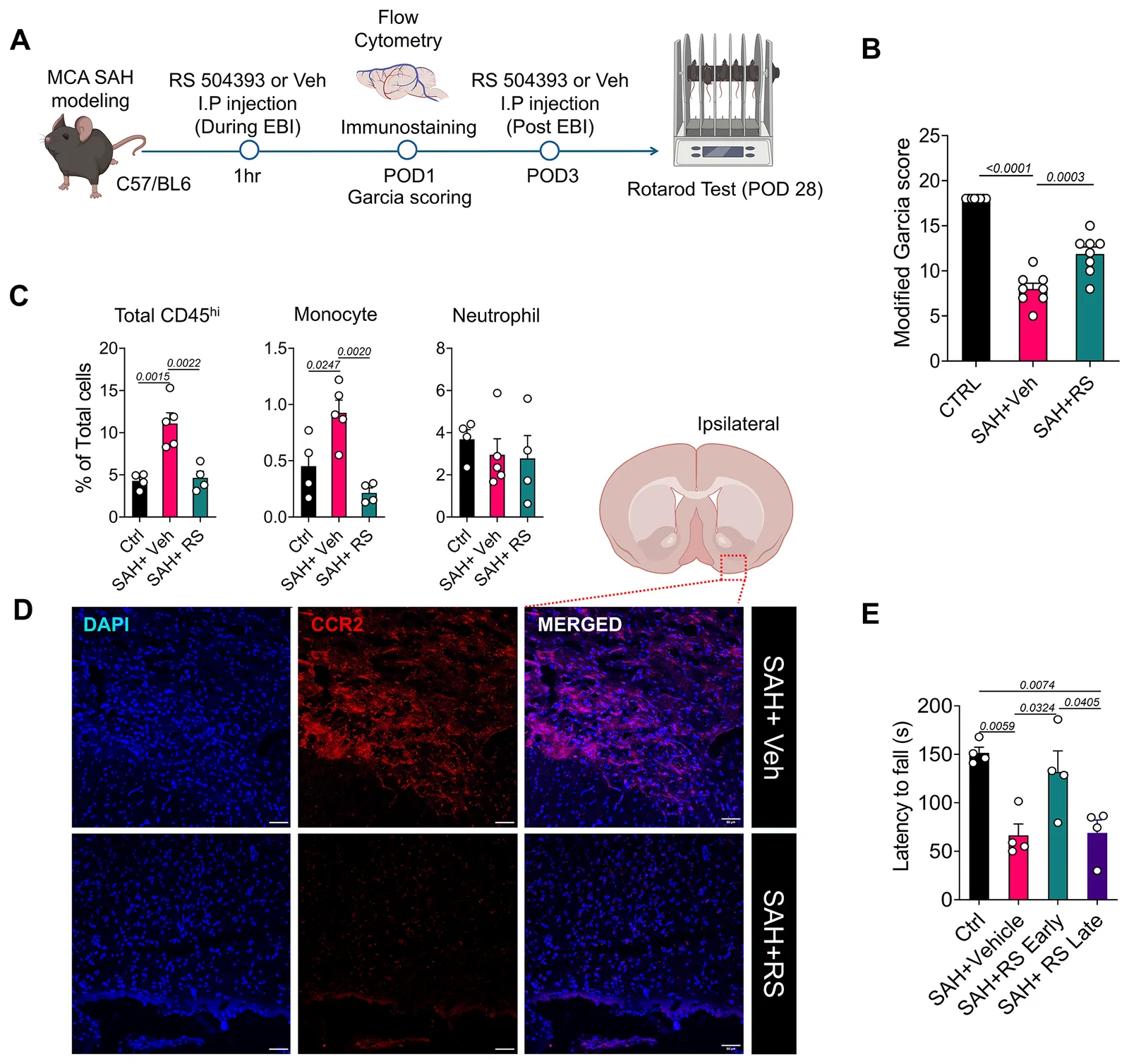
CCR2 antagonist treatment at the beginning of EBI ameliorates WT SAH mice model. **(A)** Schematics of CCR2 antagonist administration experiment after SAH induction. **(B)** Graph representing Garcia scoring of SAH mice model on POD1. **(C)** FCM analysis data of infiltrating innate immune cell population on POD1. **(D)** Representative Immunofluorescence imaging showing CCR2^+^ cells between SAH mice treated with either Vehicle or CCR2 antagonists. Area of region depicted by dotted square are shown in left figure, indicating parenchyma at perforation site. **(E)** Rotarod test result on POD 28. Panels **(B, C, E)** are presented as mean ± SEM. Panel B, N = 7–8 per group. For Panel **(C)**, N = 4–5 per group. Panel **(D)**, N = 4 per group, Scale bar = 50 μm. Panel **(E)**, N = 4 per group. Panels **(B, C, E)**, one-way ANOVA with Tukey’s multiple comparisons test was performed. Significant p-values are indicated in the graphs.

Given the improved motor outcomes observed in CCR2**^−/−^** SAH mice, we next determined whether pharmacologic CCR2 inhibition during EBI could similarly improve motor recovery in WT SAH mice (**Figure 5A**). Based on our prior findings demonstrating peak immune infiltration at POD1, we assessed modified Garcia scores and immune cell composition at this timepoint. WT SAH mice treated with CCR2 inhibitor exhibited significantly improved modified Garcia scores compared to vehicle controls, showing a pattern of recovery comparable to that observed in CCR2**^−/−^** SAH mice (**Figure 5B**; **Supplementary Video S2**). Flow cytometry analysis confirmed reduced CD45^hi^ myeloid cell infiltration, including decreased Ly6C^hi^ monocytes, in inhibitor-treated mice compared with vehicle controls (**Figure 5C**). Immunostaining of peri-perforation parenchyma demonstrated that CCR2^+^ cell accumulation was reduced in the inhibitor-treated group compared to the vehicle control group, confirming target engagement by the CCR2 inhibitor (**Figure 5D**).

To determine whether the timing of CCR2 inhibition influences long-term motor recovery following SAH, we administered the CCR2 inhibitor at two distinct time points and assessed motor performance at POD 28 (**Figure 5E**). Mice received a single dose of a CCR2 inhibitor either during the early EBI period (1h post-SAH) or after POD3 (72h post-SAH), corresponding to the post-EBI period. Rotarod testing at POD 28 demonstrated that mice receiving CCR2 inhibition during the early EBI period (RS Early) exhibited significantly improved motor performance compared with both vehicle-treated mice and mice treated after the EBI period (RS Late*)*. In contrast, RS Late showed no significant improvement compared to vehicle-treated controls (*p* = 0.900). Collectively, these findings suggest that CCR2 inhibition during the early EBI period improves motor recovery following SAH, supporting the existence of a critical therapeutic window for CCR2-targeted intervention.

### Monocyte-associated inflammatory signaling exacerbates hemin-induced neuronal injury

**Figure 6:**
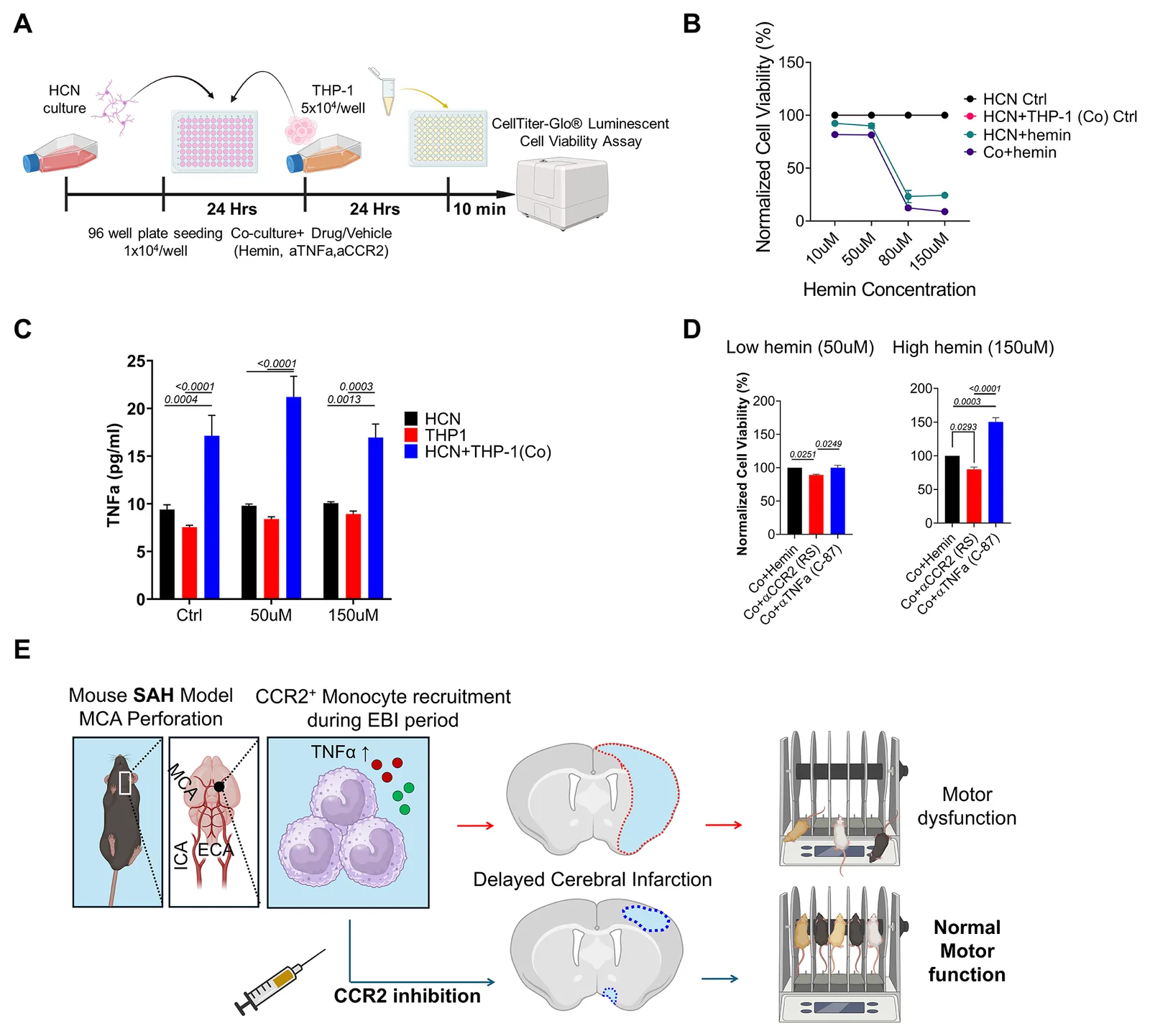
Monocyte-associated inflammatory signaling enhances hemin-induced neuronal injury *in vitro*. **(A)** Schematics of *in vitro* experimental design. Human cortical neurons (HCN) were exposed to increasing concentrations of hemin (10–150 µM) to model hemorrhagic stress. To evaluate monocyte-mediated effects, HCN were co-cultured with THP-1 monocytes in the presence or absence of CCR2 inhibitor (10 µM) or TNFα inhibitor (10 µM) along with hemin. Cell viability was assessed at 24h. **(B)** Dose-dependent cell viability following different hemin exposure. **(C)** Quantification of TNFα levels in conditioned media by ELISA under *in-vitro* experimental conditions. **(D)** Contribution of inflammatory signaling to neuronal injury. Cell viability of different co-culture+ hemin conditions was normalized to co-culture+ hemin group and compared following CCR2 or TNFα inhibition. **(E)** Proposed model: CCR2-dependent monocyte recruitment during EBI period elevates inflammatory mediators, including TNFα, which amplify secondary neuronal injury and worsen functional recovery following SAH. Panels **(B–D)** are presented as mean ± SEM. Panel **(B)**, unpaired multiple t-test with Holm–Šídák’s correction at each concentration of hemin were assessed to determine statistical significance between HCN + hemin and Co + hemin (10 µM p = 0,0004; 50 µM p = 0.0067; 80 µM p = 0.0334; 150 µM p < 0.0001). Panels **(C, D)**, two-way ANOVA with Tukey’s multiple comparisons test was used. Significant p-values are indicated in the graphs.

Given that early CCR2 inhibition improved functional outcomes and reduced inflammatory cell infiltration *in vivo*, we next sought to determine whether monocyte-associated inflammatory responses exacerbate neuronal vulnerability under hemorrhagic stress. To address this, we established an *in vitro* co-culture model using human cortical neurons (HCN) and THP-1 monocytes to model EBI-associated inflammatory conditions (**Figure 6A**).

Hemin exposure simulates hemoglobin degradation-mediated toxicity observed after hemorrhagic stroke (37, 38). HCN was exposed to increasing concentrations of hemin (10–150 μM) in the presence or absence of THP-1 monocyte co-culture, and cell viability was assessed at 24h. Hemin reduced neuronal viability in a dose-dependent manner in both HCN monoculture and co-culture conditions (**Figure 6B**). Co-culture with THP-1 monocytes resulted in significant reduction in neuronal viability compared to HCN monoculture at corresponding hemin concentrations, indicating that monocyte-associated inflammatory signaling potentiates hemin-induced neuronal injury under hemorrhagic stress.

Given that our *in vivo* SAH model demonstrated elevated CSF TNFα levels in WT mice that were reduced in CCR2^−/−^ mice (**Figure 2E**), we next examined whether TNFα-associated inflammatory signaling contributes to monocyte-associated neuronal injury *in vitro*. Notably, secreted TNFα levels were markedly elevated only under HCN-THP-1 co-culture conditions compared to HCN and THP-1 monocultures alone under hemin-treated conditions (**Figure 6C**), suggesting that monocyte-neuron interactions amplify inflammatory cytokine signaling during hemorrhagic stress. Neutralization of TNFα in the co-culture system partially restored neuronal viability under high-dose hemin exposure (150 μM), compared to hemin-treated co-culture conditions (**Figure 6D**). The protective effect of TNFα neutralization was most pronounced at high-dose hemin concentrations, supporting a contributory role for monocyte-associated TNFα in secondary injury during severe hemorrhagic stress.

Collectively, these findings suggest that while hemin-mediated toxicity serves as a primary driver of neuronal injury, monocyte-associated inflammatory responses further exacerbate neuronal vulnerability under hemorrhagic conditions. The marked increase in TNFα observed specifically in the neuron-monocyte co-culture system, together with the partial rescue achieved through TNFα neutralization, supports a model in which inflammatory crosstalk between CCR2-recruited monocytes and injured neurons amplifies secondary injury pathways during the EBI phase following SAH. These findings are further supported by our *in vivo* observations demonstrating reduced CSF TNFα levels and improved functional recovery following early CCR2 inhibition during the EBI period (**Figure 6E**).

## Discussion

Overall, our findings identify CCR2-dependent recruitment of inflammatory monocytes during the EBI period as an important contributor to neuroinflammation and secondary neuronal injury following SAH. Using both genetic deletion and pharmacologic inhibition approaches, we demonstrate that suppression of CCR2 signaling reduces monocyte infiltration, while early intervention during the EBI period attenuates pro-inflammatory cytokine production and mitigates secondary neuronal damage, resulting in improved functional recovery. Notably, the therapeutic effects of CCR2 inhibition were highly time dependent, as delayed administration beyond the EBI period failed to improve neurological outcomes. Complementary *in vitro* studies further demonstrated that monocyte-neuronal interactions enhance TNFα-associated inflammatory signaling and exacerbate hemin-induced neuronal injury. Together, these findings support an association between early CCR2-dependent monocyte recruitment, inflammatory signaling, and secondary injury after SAH, indicating a critical therapeutic time window.

The pathogenesis of SAH differs fundamentally from many other forms of hemorrhagic brain injury in that the initiating insult occurs within the subarachnoid and CSF compartments rather than the brain parenchyma (39). Unlike intracerebral hemorrhage (ICH), where inflammatory responses are largely localized to the hematoma core and perihematomal tissue, SAH results in diffuse exposure of the meningeal and CSF spaces to blood breakdown products, promoting widespread neurovascular and inflammatory activation during the EBI period. Although roles of innate immune cells have been extensively studied in models of autoimmune and neurodegenerative pathologies (40, 41), the relative contribution of infiltrating versus resident immune cells in acute neuroinflammation remains incompletely defined (42). In the present study, both genetic deletion and pharmacologic inhibition of CCR2 significantly reduced monocyte infiltration during the EBI period following SAH, supporting the concept that inflammatory monocyte recruitment in this setting is largely CCR2-dependent. Importantly, the therapeutic effects of CCR2 inhibition were highly time-dependent, as delayed intervention beyond the EBI period failed to improve neurological outcomes. These findings suggest that CCR2-mediated monocyte recruitment is associated with early inflammatory amplification following SAH and may represent a temporally restricted therapeutic target during EBI.

Reduced monocytes coincided with decreased CSF pro-inflammatory cytokine levels of IL-6 and TNFα, whereas IL-1β levels remained relatively unchanged. This differential cytokine response may reflect distinct cellular sources and temporal inflammatory dynamics during EBI. Under neuroinflammatory conditions, TNFα production is longer sustained by peripheral immune cells (43), while microglia have been reported to be prominent producers of IL-1β in models of SAH or other CNS injury (44–46). Because CCR2 signaling primarily regulates peripheral monocyte recruitment rather than resident microglial activity, the preserved IL-1β levels observed may indicate microglial responses were largely unaltered by CCR2 inhibition. In contrast, the reduction of TNFα and IL-6 supports a functional association between CCR2-dependent monocyte recruitment and amplification of the early pro-inflammatory cytokine milieu during the EBI period. These findings further suggest that infiltrating monocytes may contribute predominantly to early inflammatory propagation following SAH, while additional contributions from resident microglia, astrocytes, or other CNS-associated cells remain involved in shaping the overall neuroinflammatory response.

Our *in vitro* findings further support a model in which monocyte-associated inflammatory signaling contributes to secondary neuronal injury under hemorrhagic stress conditions. Co-culture of human cortical neurons (HCN) with THP-1 monocytes significantly exacerbated neuronal death following hemin exposure and increased TNFα production, whereas TNFα neutralization partially restored neuronal viability. Although THP-1 cells represent a surrogate model of peripheral monocytes and additional inflammatory mediators beyond TNFα are likely involved, these findings support the concept that infiltrating monocytes may amplify secondary neuronal injury through inflammatory cytokine signaling during the early stages of SAH-associated EBI.

Elevated IL-6 in CSF has been associated with disease severity and prognosis in SAH (39, 47, 48), although its precise functional role remains complex. Our findings collectively support a model in which CCR2-dependent monocyte recruitment enhances these pro-inflammatory cytokine signaling, including TNFα and IL-6, thereby amplifying secondary injury during the EBI period. Our findings highlight the temporal importance of CCR2 signaling during the EBI period and demonstrate that early, but not delayed, CCR2 inhibition mitigates secondary injury and improves functional recovery.

The involvement of CCR2 signaling in SAH and other hemorrhagic strokes has been reported by other previous studies. Inflammatory alterations within choroid plexus following SAH have been reported, including upregulation of innate immune receptors and inflammatory mediators that may contribute to amplification of CNS neuroinflammatory responses, supporting the concept of early immune activation at CNS border regions contributing to secondary injury after SAH (49). Also, in intracerebral hemorrhage (ICH), where inflammatory infiltration predominantly occurs within the perihematomal parenchyma, CCR2 deficiency is associated with early neurological recovery (50). While studies indicate association of CCR2 signaling and its importance in affecting immune recruitment and neurological recovery following SAH, we cannot exclude additional effects of CCR2 inhibition beyond modulation of monocyte trafficking. Recent work has suggested that CCR2 expressions may also be upregulated within neuronal populations after SAH and that pharmacologic CCR2 inhibition attenuates neuroinflammation and neuronal apoptosis, potentially through modulation of PI3K/Akt signaling pathways (27). This raises the possibility that CCR2 signaling may influence both immune cell recruitment and cell-intrinsic survival pathways, suggesting that the therapeutic effects of CCR2 inhibition may be multifaceted.

Building upon these findings, our study further supports CCR2 signaling as an important contributor to hemorrhagic brain injury and suggests a role for early CCR2-dependent monocyte recruitment during the EBI period as a “temporally restricted contributor” to inflammatory amplification following SAH. Using both genetic CCR2 deletion and pharmacologic inhibition approaches, we demonstrate that therapeutic benefit was highly dependent on intervention timing, as delayed CCR2 inhibition beyond the EBI period failed to improve long-term functional outcomes. In addition, flow cytometric profiling and cytokine analyses demonstrated that reduced CCR2-dependent monocyte infiltration was associated with attenuation of TNFα- and IL-6-associated inflammatory signaling, supporting a functional contribution of infiltrating monocytes to secondary injury progression after SAH. While our study highlights CCR2 involvement in time-dependent peripheral immune cell recruitment, further investigations using cell-specific deletion strategies will be necessary to delineate the relative contributions of immune versus neuronal CCR2 signaling in secondary injury after SAH.

TUNEL staining revealed significant differences in neuronal apoptosis between SAH groups, supporting the conclusion that CCR2 inhibition attenuates secondary neuronal injury during the EBI period. While CCR2 inhibition would not be expected to mitigate primary insults such as increased ICP or hemin stress, the observed reduction in neuronal cell death suggests that attenuation of CCR2-associated inflammatory responses may contribute to partial neuroprotection. These findings are consistent with the concept that early inflammatory amplification contributes to secondary injury progression following SAH.

Previous studies showed early anti-inflammatory interventions, including ibuprofen, can attenuate neuronal damage and vasospasm (51–53), although therapeutic effects were limited. This highlights the importance of the EBI period as a critical therapeutic window. Inflammatory signaling during this phase must be carefully balanced, as an initial pro-inflammatory response may be necessary for vascular repair and debris clearance (54, 55). Hence, broad immunosuppression may disrupt essential recovery processes. Selective modulation of CCR2-dependent monocyte recruitment appears to preferentially reduce excessive cytokine production, including TNFα and IL-6, without globally suppressing resident immune responses. This may explain the functional improvements observed in our study, suggesting that controlled attenuation of neuroinflammation, rather than complete immune suppression, may be essential for a more effective therapeutic strategy.

Consistent with reduced neuroinflammation and neuronal death, both genetic CCR2 deletion and early pharmacologic CCR2 inhibition improved motor recovery, as reflected by enhanced Garcia and rotarod performance. Notably, no significant differences were observed in cognitive performance. Taken together, our study provides evidence that CCR2 inhibition mitigates the pro-inflammatory effects of infiltrating monocytes, supporting CCR2 as a potential therapeutic target for improved motor recovery following SAH. Clinical studies report variability in cognitive outcomes following SAH (56, 57), suggesting that CCR2-mediated inflammatory mechanisms predominantly influence early motor circuitry or acute injury severity rather than long-term cognitive domains. Importantly, peripheral monocyte-related indices have been identified as significant predictors of patient outcomes post-SAH (58), supporting the clinical relevance of our findings. Together, these results corroborate the concept that CCR2-dependent monocyte recruitment contributes to early secondary injury and that timely modulation of this pathway may improve motor recovery following SAH.

Several limitations exist in this study. First, the endovascular perforation model carries inherent variability in hemorrhage severity, which cannot be controlled precisely; we were unable to determine whether CCR2 inhibition brought a stable therapeutic effect across different SAH grades. In addition, while our data supports a role for CCR2 in regulating inflammatory monocyte infiltration during the early injury phase, further studies are required to assess severity-dependent effects and cell-specific targeting strategies. Our *in vitro* experiments utilized human cortical neurons co-cultured with the THP-1 monocytic cell line to model monocyte–neuron interactions under hemin-induced stress. Although this system enabled mechanistic evaluation of TNFα-mediated effects, THP-1 cells may not fully recapitulate primary human monocyte heterogeneity, and the simplified co-culture lacks other components of the neurovascular unit. The partial rescue observed with TNFα neutralization further suggests that additional inflammatory mediators likely contribute to neuronal injury.

In conclusion, our findings demonstrate that early inhibition of CCR2 during the EBI period reduces monocyte infiltration, attenuates pro-inflammatory cytokine signaling, limits neuronal cell death in the motor cortex, and improves functional recovery after SAH. These results are consistent with a contributory role for CCR2-dependent monocyte recruitment in secondary injury and support temporally targeted CCR2 modulation as a potential therapeutic strategy for improving outcomes following subarachnoid hemorrhage.

## Data Availability

The original contributions presented in the study are included in the article/**Supplementary Material**. Further inquiries can be directed to the corresponding authors.
