# Supplementary material for: Early CCR2-dependent monocyte recruitment defines a critical neuroinflammatory window after subarachnoid hemorrhage
Source: Front Immunol. 2026 Aug 14;17:1888359. doi: 10.3389/fimmu.2026.1888359 (PMC13522166; doi:10.3389/fimmu.2026.1888359)
Supplement: Supplementary file 1 [file SupplementaryFile1.docx]

**Supplementary Materials**

**Methods**

**PLX administration**

Mice subjected to PLX treatment were given PLX 3397 infused Purina chow (600ppm) over 7 days prior to SAH modeling. Then, modified Garcia scoring was performed in accordance with table 1 on POD 1 and 3.

**Morris Water maze**

To test the memory difference between two SAH outcomes, mice were selected based on the modified Garcia score to minimize mobility leading to a problem. Any mice on POD5 Scoring below 10 were excluded from the experiment, and any mice that had a score of 16 or above were considered to have lighter severity and were excluded. Water maze training and test was used following the protocols written by Vorhees et al., 2006^1^ with slight modifications to time. Mice were placed in 120cm diameter swimming maze filled with water and white paint, with water temperature kept at a range of 22-23°C. success latency was measured based on the initial time it landed on platform and continued to stay for 15 seconds or more. Then, a probe test was done by removing the platform and video recording was performed for 60 seconds. The videos were analyzed using Ethovision software to determine how often mice crossed the locations of the platform, to determine its spatial memory. Quadrants were divided based on platform as the target quadrant.

**Immunohistochemistry**

All samples used for immunohistochemistry’s pre-preparation were the same as TUNEL staining, with exceptions to one antigen retrieval step after deparaffination. Antigen retrieval solution made by Sodium Citrate (Sigma, S1804), with PH adjusted to 6.0, was warmed up to 86-90°C, and slides were placed in solution for 30 minutes prior to Staining process. Slides were rinsed with PBS three times and to remove endogenous peroxidase response, slides were incubated in 0.3% peroxide in PBS for 15 minutes. Then after one quick rinse with PBS, slides were blocked for 30 minutes using 3% BSA 0.3% Triton-X in PBS. Afterwards, Iba-1 antibody (Abcam, ab178846) was diluted in antibody dilution solution (1% BSA 0.1% Triton-X in PBS) and slides were incubated in it over night at 4°C. Then slides were washed in PBS three times 5 minutes each, and Secondary anti-rabbit HRP (DAKO, K400311-2) was applied for 1 hour at room temperature. DAB substrate Kit (Dako, K346711-2) was then applied to the tissue for staining. Beyond this point, starting from hematoxylin counter staining, rest of the procedures followed the same procedure as the TUNEL staining process.

**Supplementary Figures and Tables**

**Supplementary Table 1. Garcia Scoring table.** Table showing test performed and scoring standard based on behavioral pattern.

**Supplementary Table 2. Statistical Analysis table for applicable main Figures.** Test type, Relevant Statistics, P-value of the test done, and effect size is written in accordance to the test performed on relevant figures.

**Figure 1. Cytokine levels within the SAH Brain Lysates.** Cytokines were obtained by lysating brain excluding cerebellum and brainestems.

*n=6 per group. Graphs are* *presented as mean ± SEM. One-way ANOVA with Tukey’s multiple comparisons test was used. Significant p-values are indicated in the graphs.*

**Figure 2. Additional Behavioral test.** A) Garcia scored on POD 1 and 3 for comparison of WT SAH vs PLX treated SAH mice model. *N=5 per group, One-Way ANOVA, ** = P<0.01* B) Schematic figure of MWM test for SAH mice model. C) Representative images of analyzed mice swimming track from MWM probe test. The last 30 seconds of movement track (Left) and Heatmap (right) are shown. D) Analyzed graph showing latency to land on platform during training (top) and time spent during probe test in different quadrant (bottom). *n=5 per group. Figures B and D are presented as mean ± SEM. Two-way RM-ANOVA, with* *Holm-Šídák's multiple comparisons test performed. Significant p-values are indicated in the graphs.*

**Figure 3. Immunohistochemistry and additional TUNEL staining for SAH model.** Representative Iba-1 Staining result within the motor cortex, obtained by serial section of the sample sample shown in Figure 4. B) Additional TUNEL staining of section beyond the samples shown in Figure 4, within Dentate Gyrus. C) Representative Immunofluorescent staining of TUNEL with anti NeuN to determine neuron-specific cell death. Scale bar = 50 μm. NeuN *N=4 per group*

**Figure 4: TUNEL & NeuN Co-localization Analysis**. A) Representative images showing TUNEL and NeuN staining used for co-localization analysis. *Scale bar = 50 μm.* B) Quantification of TUNEL/NeuN co-localization. In sections with intense TUNEL staining, NeuN labeling was markedly reduced, limiting reliable assessment of TUNEL/NeuN co-localization. Therefore, representative images from adjacent serial sections of the same animal of same cortical region were used, in which both TUNEL and NeuN signals could be adequately visualized, and were used for image presentation and co-localization analysis. *n= 2~3 per group. Figure B is presented as mean ± SEM. One-way ANOVA with Tukey’s multiple comparisons test was performed. Figures C-D are presented as mean ± SEM. Significant p-values are indicated in the graph.*

**Supplementary Video 1. Garcia Climbing test video of WT vs CCR2^-/-^.** Representative Garcia climbing test of Ctrl and SAH group for WT and CCR2^-/-^

**Supplementary Video 2. Garcia Climbing test video after CCR2 inhibition.** Representative Garcia climbing test of Ctrl, SAH+ Vehicle, and SAH+ RS (CCR2 inhibitor) respectively.

**Supplementary References**

1.	Vorhees CV, Williams MT. Morris water maze: procedures for assessing spatial and related forms of learning and memory. *Nat Protoc*. 2006;1:848-858. doi: 10.1038/nprot.2006.116
